# Spinal Cord Microglia in Health and Disease

**DOI:** 10.32607/actanaturae.10934

**Published:** 2020

**Authors:** E. A. Kolos, D. E. Korzhevskii

**Affiliations:** Institute of Experimental Medicine, St. Petersburg, 197376 Russia

**Keywords:** microglia, spinal cord, embryogenesis, immunohistochemistry, multiple sclerosis, ALS, aging

## Abstract

The review summarizes data of recent experimental studies on spinal microglia, the least explored cells of the spinal cord. It focuses on the origin and function of microglia in mammalian spinal cord embryogenesis. The main approaches to the classification of microgliocytes based on their structure, function, and immunophenotypic characteristics are analyzed. We discuss the results of studies conducted on experimental models of spinal cord diseases such as multiple sclerosis, amyotrophic lateral sclerosis, systemic inflammation, and some others, with special emphasis on the key role of microglia in the pathogenesis of these diseases. The review highlights the need to detect the new microglia-specific marker proteins expressed at all stages of ontogeny. New sensitive and selective microglial markers are necessary in order to improve identification of spinal cord microgliocytes in normal and pathological conditions. Possible morphometric methods to assess the functional activity of microglial cells are presented.

## INTRODUCTION

Microgliocytes are one of the most mysterious spinal cord types of cells that are significant for understanding the pathogenesis of neurological diseases. This is due to the crucial role they play in the regulation of pain sensitivity and pathological responses leading to demyelination and neurodegeneration. The first description of microglial cells and introduction of the term “microglia” was made by the Spanish researcher Pio del Rio Hortega 100 years ago, in May 1919 [1]. He characterized the microglial cells of the gray matter of the brain and suggested the important role they play in the protective mechanisms of the nervous tissue. Since the studies by Pio del Rio Hortega and until recently, the functional significance of microglia has been associated with the local immune response to injury. However, according to modern concepts, microglia are able to perform other functions and the range of these functions can vary in different periods of ontogenesis (before birth, after birth, and during aging).

Microglial cells are a special type of tissue macrophages of the central nervous system (CNS) and are often considered one of the forms of specialized mononuclear phagocytes [2-4]. Microgliocytes are the main cells of the immune response in the CNS which play an important role in homeostasis of the nervous system during its formation, both in normal conditions and in pathology, by ensuring the regulation of inflammatory processes. These cells are able to protect the CNS structures from damage by phagocytosis, presentation of antigens, and cytokine secretion [5]. The role of microglia in the CNS in the absence of damage and during the development of the spinal cord and the brain remains the subject of active research and discussions. Microgliocytes were found to be involved in the regulation of neuron development, functioning, and death. Microglial cells are also known to provide trophic support for neurons and play an important role in synaptic remodeling and synaptogenesis; they are also directly involved in the formation and reorganization of neural networks [2, 6, 7]. Microgliocytes are believed to be able to perceive and regulate local neuronal activity [8], as well as secrete neuroactive substances [9, 10]. In recent years, there has been renewed interest in studying microglia [11]. Meanwhile, data on various aspects of the biology of cerebral microglia are regularly summarized in numerous reviews, while a sufficiently large body of research on spinal cord (SC) microglia still requires systematization and critical re-evaluation.

The aim of the current study was to summarize recent data on spinal microglia during development, in pathology, and in aging. The data are preceded by a brief summary of classical and modern methods for detecting microglia.

## METHODS FOR IDENTIFICATION OF MICROGLIOCYTES

For a long time, the only universal approach to detecting microglial cells was the silver impregnation method. Various versions of the impregnation technique are known, the first of which was developed by Rio Hortega P. [1] and could be utilized only for frozen sections. There are the Miyagawa and Alexandrovskaya methods that were also developed for frozen-tissue sections, and the Belezky–Stern method used for celloidin sections [12-16]. These techniques cannot be used for paraffin sections of the SC, since they are laborious, difficult to reproduce, and not selective enough (beside the microglia, oligodendroglial cells are also stained).

At the end of the twentieth century, more selective methods for the detection of microglia were developed based on the use of immunocytochemical approaches. Their reproducibility was significantly higher than that for classical impregnation techniques. In addition, it became possible to use fluorescence methods, including confocal microscopy, for the visualization of microglia. Nevertheless, immunohistochemical detection of CNS microgliocytes remains a rather complicated process, due to the species-specificity of most of the antibodies used and the similarity of the set of markers for microgliocytes and the cells of the macrophage lineage, which belong to a cell population different from microgliocytes.

Microglial elements of the SC and the brain express a wide range of markers characteristic of macrophages and blood monocytes, including the glycoproteins F4/80 and CD68, proteins of the major histocompatibility complex class II (MHCII), integrin CD11b, the macrophage colony-stimulating factor 1 receptor (CSF1R), and the proteins CD115 and Iba-1 [17]. Adult SC and brain microgliocytes, unlike most other tissue macrophages, express high levels of the fractalkine receptor CX3CR1 [18].

The protein lba-1 (Ionized calcium-binding adapter molecule-1) is a microglial marker most commonly used for studying spinal microglia in normal and pathological conditions. This protein can interact with actin molecules and is involved in the reorganization of the cytoskeleton and the changes in the configuration of the cell membrane during phagocytosis [19]. The Iba-1 protein is fairly evenly distributed both in the cytoplasm and in the microglial processes [20]. It allows one to identify all known morphological types of microglia, as well as detect and analyze the complex process structure of cells [21, 22]. An unusual property of Iba-1, which has not yet been adequately explained, is its intranuclear concentration at certain loci not associated with heterochromatin and nucleolus [23].

Recently, a microglia-specific immunohistochemical marker has been proposed: the transmembrane protein TMEM119 (transmembrane protein 119). The function of this protein is not well understood. However, it has been established that, unlike most other microglial markers, it is not expressed by macrophages and other types of immune cells. In addition, it is also absent in nerve cells, astrocytes, and oligodendrocytes. This marker allows for selective detection of microgliocytes in the postnatal period, in normal condition, and in SC pathology [24, 25, 26]. However, TMEM119 is absent in immature microglia in the prenatal and early (prior to P14) postnatal periods [25].

Identification of microgliocytes in the SC of mice using antibodies to the transmembrane sialic acidbinding Ig-like lectin (Siglec-H) revealed that this marker allows for selective detection of microglial cells both at the stage of embryonic development and in the postnatal period. This marker also allows one to study the cell morphology. In addition, Siglec-H is also expressed in activated microglial cells in trauma and inflammation. Siglec-H can be used as a histological marker to distinguish between the CNS microglia and monocytes penetrating into the SC [27]. A transcriptome analysis showed that the Siglec-H gene is the third most expressed gene (out of the 29 identified) that allows one to distinguish microglia from monocytes and macrophages [28].

It has been established that the P2Y12 receptor (P2Y12R) to adenosine-5’-diphosphate (ADP) is expressed in inactivated (resting) microgliocytes. The content of this protein on the surface of activated microglia rapidly decreases [29, 30, 31]. P2Y12 allows one to identify and study the complex organization of the processes in microgliocytes of intact SC. P2Y12R expression is not observed in neurons and astrocytes of the SC, while platelets are immunopositive for this receptor [32]. Immunohistochemical detection of P2Y12 and a change in its expression can potentially have a prognostic value in neuroinflammatory conditions. Modern studies indicate that, unlike classical microglial markers (Iba-1, CD68, and MHCII), P2Y12 is present on microgliocytes but is absent in perivascular and meningeal macrophages. In addition, P2Y12 can be used as a microglial marker both in the pre- and postnatal periods [31].

Immunohistochemical methods are indispensable in studying human microglia, while in experimental studies more complex genetic methods for marking microgliocytes can be used. For this, transgenic lines of animals are usually generated. In such models, an easily detectable molecule, such as the green fluorescent protein (EGFP), is expressed under the control of the promoter of the microglia-specific gene. To date, several mouse lines which contain loci of microglia signature genes (Iba-1, Runx1, Csf1r, Lyz2, Itgam, Sall1, and CX3cr1) for fluorescent cell labeling are available [33-36]. However, the use of transgenic animals of the above-mentioned lines is complicated by the fact that it is rather difficult to separate activated microgliocytes from functionally similar cells such as blood monocytes, perivascular macrophages, meningeal macrophages, and choroid plexus macrophages [37]. Meanwhile, the obvious advantage of genetic labelling of microglia is that dynamic intravital imaging can be performed.

## I. MICROGLIA IN HEALTH

Initially, the spinal cord anlage (as well as the brain anlage) in mammals and humans are devoid of microglial cells. Discovery of this fact contributed to a long scientific discussion on the origin of microglia [38]. The first assumption was that microglia originate from mononuclear elements of the blood [1, 39]. Later, other concepts of microglia origin also appeared [40-42]. In the early 1990s, it was widely believed that they are of mesenchymal origin from precursors of the monocyte-macrophage lineage: hematopoietic stem cells penetrating the CNS during embryogenesis [43]. However, it was noted that the first precursors of microglia penetrate the CNS before the formation of the unitary vasculature and the establishment of definitive bone marrow hematopoiesis. In other words, the colonization of embryonic CNS by microglial precursors precedes the appearance of blood stem cells, which indicates an alternative origin of microglial progenitors. This fact may be an indication that they originate from a given subset of mesodermal cells not directly associated with the monocytic lineage [44]. Indeed, recent studies have shown that microglia differ from tissue macrophages, which are derivatives of monocytes and, accordingly, descendants of blood stem cells. Currently, several genes have been identified whose activity allows one to separate microgliocytes from tissue macrophages [28, 45].

In recent decades, compelling evidence in favor of the theory that microglial cells originate from erythro- myeloid progenitor cells of the yolk sac colonizing the CNS in the early stages of embryonic development has appeared. This theory was proposed in 1999 [46] and later confirmed experimentally [34, 47-51]. It was shown that F4/80+/CD11b+ microglial precursors are present in the yolk sac of the mouse embryo already at the E8 stage. In a developing CNS, these cells are detected starting at E9, while definitive hematopoiesis in mice begins at the stage E10.5 in the aorta-gonadmesonephros region. The first hematopoietic stem cells found in this area migrate to the liver and bone marrow, where hematopoiesis proceeds [46, 52].

Modern studies report that the penetration of microglial elements into the SC tissues occurs in two stages. The first stage, during which microglial cells reach the SC through the developing vasculature, corresponds to E8–E9, and the second stage corresponds to E11.5–14.5. The first stage is directly related to the development of the perimedullary vasculature [53]. At the second stage, an increase in the number of microglial cells in the already formed vasculature takes place. After the blood–brain barrier (BBB) closes, microglial cells that have entered the CNS become a self-sustaining cell population [48, 50, 54]. It is important to note that, simultaneously with the second wave of microglia migration (E11.5), active synaptogenesis and formation of neural networks take place in the spinal cord anlage [55, 56]. Some studies report that precursors of microglial cells migrate through the central canal to the ventricular zone, where they actively proliferate, which explains the rapid increase in the number of SC microgliocytes at E12.5 [57]. Similar processes are also observed in chicken embryos [58]. A study of the SC embryogenesis in mouse and chicken revealed an accumulation of microgliocytes in the dorsal region of the SC anlage at E12.5 and E3.5, respectively. It is known that afferent nerve fibers of the dorsal root ganglion (DRG) begin to grow into embryonic SC at E11.5 and E3 in mice and chicken, respectively [59]. Therefore, it is logical to assume that projections of afferent DRG neurons can serve as a mediator for the penetration of microglia into the dorsal part of the gray matter of the SC [57, 58].

**Morphological and cytochemical features of microglia**

During the period of embryogenesis, microgliocytes of the SC and brain undergo several stages of development. In the early stages of prenatal development, microglial cells have an amoeboid shape, as well as immunological, histochemical, and morphological features they share with tissue macrophages. These cells have a rounded shape with short, thick processes [60, 61]. During development, the processes of amoeboid microgliocytes lengthen, branch, and the cells acquire the shape characteristic of ramified microglia. It was demonstrated that a portion of the SC microgliocytes of mouse embryos display several thin processes by the stage E12.5, which become branched by E15.5 [57].

Reports on the assessment of the SC response to pathological stimuli note the presence of two morphological functional types of microgliocytes, as well as intermediate forms [62-65]. Resting (ramified), activated (amoeboid) microgliocytes, and transitional forms are traditionally distinguished in the SC. The first type are stationary cells characterized by a small cell body with several thin branching processes, which actively scan the environment, thus gathering information about the tissue state and regulating the state of synapses and neural network development. In such cells, the branched processes quickly lengthen and contract in random order, thus occupying various spatial areas (cell territories) with minimal overlap. A similar activity by microglial cells is commonly referred to as basal (main) motility. The cells are assumed to be incapable of phagocytosis and do lack the diversity of surface receptors necessary for antigen presentation.

In response to damage, microglia quickly project their processes towards the site of the danger signal (directional motility) [63, 66]. In pathological conditions, SC microglia change their morphological and molecular properties through long-term transformations called microglial activation. When microglia are activated in response to a SC injury, a reduction in the number and extent of cell processes is observed. Ultimately, the cells acquire an amoeboid form, which is accompanied by the production of bioactive molecules, thereby contributing to the activation of inflammatory reactions and protection against damaging agents [62-65]. Amoeboid microglia are known to be highly motile phagocytic cells involved in antigen presentation. The process involved in this transition is quite complicated. During activation and inactivation of microglia, transient forms (hypertrophied and bushy cells, uni- and bipolar cells) with one or two thickened branched processes are detected [63, 67]. Such microgliocytes are considered to be cells with various degrees of activation which are present in CNS injuries and can also be detected in intact SC [67]. Despite the fact that various morphological changes in microglial cells are now described in detail, in most cases, the relationship between a particular morphology and the functionality of microglia is not particularly convincing [68]. Thus, it is rather difficult to judge the degree of microglial activation using just morphological characteristics. Nevertheless, numerous studies describing the characteristics of the microgliocyte response to damage contain data on the distribution of various morphological types of these cells in the SC. For instance, one large-scale study describes various types of microglial cells in human SC at different stages post-injury [69]. However, the work does not present the morphological characteristics of the cells of intact SC. Determination of the structural features of microglia forms would be more acccurate when taking into account the topography and when performing a comparison with the control. An example of such an approach is a study performed on a biological model of nerve damage [63]. The distribution of various morphological types of microgliocytes was studied on Rexed’s laminae. The number of hypertrophied and branched microglial cells was shown to increase significantly compared to the contralateral side after nerve lesion in the surface layers of the SC dorsal horn (Rexed’s laminae I–II) and in the region of Rexed’s lamina IX [63]. A number of studies have shown that activation of microgliocytes of the SC gray matter can occur without a significant change in the type of process branching, while the cells in the white matter change their shape [70, 71].

Activated SC microglia can acquire various immunophenotypes in response to various external stimuli [72-75]. Two functional categories of activated microgliocytes are known. They are the pro-inflammatory M1 phenotype (classical pro-inflammatory neurotoxic phenotype), which is characterized by the production of pro-inflammatory and neurotoxic mediators, and the alternatively activated M2 phenotype (antiinflammatory neuroprotective phenotype) involved in tissue repair and remodeling via the secretion of anti-inflammatory mediators [73, 76]. M1-like microglia typically express IL-1β, IL-6, IL-12, IL-23, tumor necrosis factor α (TNF-α), inducible nitric oxide synthase (iNOS), and CD16/32, while M2-like microgliocytes express these markers at a relatively low level and IL-4, IL-10, IL-13, BDNF, arginase 1 (Arg1), and mannose receptor C- type 1 (MRC-1), at high levels. However, it should be noted that, in the early postnatal period, CNS microglia express both M1 and M2 markers [74, 77, 78]. Short-term or moderate damaging effects are believed to guide microglia to the neuroprotective phagocytic phenotype. These cells secrete growth factors associated with remyelination and promote regeneration. Intense acute or chronic activation promotes the transition of microglia to the neurotoxic phenotype. These cells produce reactive oxygen species, nitric oxide (NO), proteases, and pro-inflammatory cytokines such as IL-1, IL-6, and TNF-α. Compression injury to the SC revealed a transient reaction of type M2 microglia, which, through their neuroprotective properties, can activate recovery processes. However, cells of the mixed M1/M2 phenotype are also present in the lesion area, which indicates the lability of microgliocyte phenotypes and instability of the balance between neuroprotection and neurotoxicity. As a result, the activity of type M1 microgliocytes can lead to the loss of neurons and demyelination, despite the presence of neurotrophic factors [79].

The presented classification of microglia immunophenotypes is rather arbitrary. The processes of classical and alternative activation are well studied only for macrophages [80] and are often extrapolated to microglia without sufficient grounds. M1 and M2 macrophage groups were identified in *in vitro *studies of isolated cells under the influence of model factors. The theory of macrophage activation has been developed for a population of cells deriving from blood or bone marrow monocytes that migrate into infected, injured, or tumor tissues. Microglia, on the contrary, are residential phagocytic cells of the nervous system of non-monocytic origin. *In vivo *states of M1 and M2 cells of macrophage lineage are never found separately from each other. Numerous studies have demonstrated that known markers of activated states are typically co-expressed in individual cells [77, 81]. Thus, the widely used markers of activated M1/M2 macrophages do not adequately describe the transcriptional profile of microgliocytes and cannot confirm that the cells belong to these functional groups *in vivo*.

The functional activity of microglia correlates with its structural organization. Therefore, a change in the size and shape of microgliocytes, as well as in the organization of their processes, can serve as an indicator of an impaired blood-SC-barrier. Moreover, a change in the morphological type is not necessarily observed in such a case. Structural changes in cells can be identified visually, which is quite subjective, or can be quantified using parameters such as the degree of process branching, changes in body shape, and size. Sensitive quantitative methods for detecting insignificant, visually undetectable responses by microglia to CNS damage can be used to determine pathological changes in individual CNS regions and identify loci of unknown pathology. Computer morphometry methods accelerate and facilitate the measurement of a number of the quantitative parameters of microgliocytes. They can be used to quantify the stages of the pathological process, which should contribute to an adequate prediction of the outcome of nervous tissue repair. Structural changes in microgliocytes are most often quantified using traditional morphometric methods and the basic ImageJ software (https:// imagej.nih.gov/ij/download.html) or its modified analogues (for instance, Fiji – http://fiji.sc; ImageJFX – http://www.imagejfx.net) with various plugins, such as AnalyzeSkeleton (2D/3D), Fractal Analysis/ FracLac, and NeurphologyJ [82-85]. By using these plugins, it is possible to summarize the quantitative characteristics of a microgliocyte structure, such as the location of the process endpoints, the degree and extent of branching, as well as the shape parameters of the cells.

Combined protocols for assessing changes in microgliocyte morphology can be applied in determining the reactivity of this cell population in intact and injured nerve tissue. For a quantitative analysis, both fluorescent dyes and the immunoperoxidase reaction can be used. Usually, a morphometric analysis is performed after a immunohistochemical reaction with Iba-1 [84]. Fractal analysis allows one to determine parameters such as cell density, coverage, roundness, completeness, and elongation (the ratio of the long and short axes of the cell body). Multifractal scaling allows one to identify microglia in transition states between the branched and activated forms [67, 84]. In order to extract additional information on the function of microgliocytes that constantly move their processes in an intact CNS, as well as to assess the changes in the morphological features of the cells in response to damage and aging, a Sholl analysis is used [86, 87]. This is a quantitative method used to study the radial distribution of the processes of microgliocytes. By using this method, one can gather formal numerical data on the number of cell processes and the nature of their branching. In addition, the analysis can utilize the total length of the processes, the surface coverage area, the number of branch points, as well as the branch order [87, 88, 89].

**Functions of microglia**

To date, a large amount of data has been collected on the role of CNS microglia in physiological conditions. It was initially believed that intact microgliocytes are resting immune cells capable of activation only in response to pathological changes occurring in the CNS [90]. However, numerous studies have shown that the microgliocytes in the brain and SC of an adult are highly dynamic cells that are constantly controling the intercellular environment even in an intact CNS [91]. Moreover, microgliocytes have the ability to express receptors for a large number of neurotransmitters [8], including acetylcholine, GABA, glutamate, ATP, etc. It has been shown that neurotransmitters can affect microglia *in vitro *thus leading to changes in membrane potential and intracellular calcium concentration, causing cytokine release and altering general cellular motility [8, 92].

The functional significance of microglial cells during early embryonic CNS development remains unclear due to a lack of information on the features of SC colonization with microgliocytes in relation to specific stages of development and the processes of functional neural network formation. The occurrence of microglia in a developing SC during embryonic CNS development correlates with the presence of apoptotic cells [57]. Association of microglia and neurons in physiological cell death was established in different CNS regions, including the SC [57, 58, 61]. A study focused on embryonic CNS development showed that microglia can direct the transition of cells to apoptosis through the expression of various factors. Thus, the microglial tumor necrosis factor-alpha (TNF-α) can initiate the death of motor neurons in a rodent embryonic SC [93].

Throughout the entire period of embryogenesis, microgliocytes are closely interconnected with the developing vessels [57, 94]. The hypothesis of the penetration of microgliocyte precursors in the developing SC through the blood vessels is widely discussed [34, 57]. There are data indicating that microglia are absent in the CNS of mutant mouse embryos with a defect in the development of the cardiovascular system when hemocirculation is absent [34]. Nevertheless, microglia appear in the CNS even before the stage of brain anlage vascularization [57, 95]. This suggests that early microglia precursors colonize the CNS independently of blood vessels. Furthermore, they can affect angiogenesis as well. In model conditions, microglia quickly migrate to the developing vessels and exhibit angiogenic activity [96].

The mature nervous system is characterized by the presence of a system of precise neuronal connections. However, neurons retain the ability to form new synaptic contacts, thus modifying the synaptic network. Formation of a large number of excess synapses takes place in a developing nervous system. During synaptic pruning, many synapses are eliminated: the neural network becomes optimized and simplified. Data from numerous studies indicate that interactions between microglia and developing synapses play an important role in the elimination and maturation of synaptic components in a developing CNS. It is also assumed that microglia can independently initiate synaptic elimination and regulate synapse maturation [97, 98].

In the adult SC, microgliocytes also perform the function of neural network remodeling through direct interaction with synaptic elements [97-100]. It has been established that cell processes dynamically interact with axon terminals and dendritic spines with an average frequency of about one microcontact per hour [91]. The duration of contacts between microglial processes and presynaptic boutons is approximately 5 min [91]. It is noteworthy that microglial processes are under the control of neuronal activity and can simultaneously interact with both presynaptic and postsynaptic elements, as well as with perisynaptic astrocytes [101]. Reduced neuronal activity due to the suppression of sensory effects or a decreased body temperature leads to the retraction of microglial processes and a decrease in the frequency of contacts between microglia and synapses [102]. It was shown that microglia can absorb remodeled synaptic elements by phagocytosis. In addition, in a mature CNS, microglia can modulate the plasticity of neural circuits via the paracrine pathway.

Under conditions of gravitational unloading, an increase in the number of Iba1 immunopositive microgliocytes in the dorsal horns of the SC of experimental animals was observed [103], which is an indication of a response by microglia to a decrease in the afferent stimulation of neurons in the corresponding region. The observed increase in the number of microgliocytes in the region of the central canal can be associated both with the heterogeneity of microglia in this region [104] and with possible changes in the dynamics of cerebrospinal fluid, to which subependymal microglia are able to respond to the presence of processes that directly interact with the cerebrospinal fluid [105].

## II. MICROGLIA IN SPINAL CORD PATHOLOGY

The range of pathological processes that develop in a SC in which the response of microglial cells changes is wide. In the current review, special attention is paid to such severe and disabling pathological conditions as spinal cord injury, neurodegeneration, as well as age-related pathology of the SC.

**Microglia in spinal cord injury**

Spinal cord injury (SCI) is a serious pathological condition that is accompanied by cell damage and death, hemorrhage, inflammation, tissue edema, ion imbalance, axon loss, demyelination, activation of immune cells, astrogliosis, as well as reorganization of the vascular system and neural circuits [106]. In SCI and other pathological processes, resident microglia quickly respond to changes in the microenvironment by releasing specific cytokines, leukotrienes, and prostaglandins [107]. Under certain circumstances, microglial cells can exert a neuroprotective effect through the synthesis of neurotrophic factors [108, 109]. The most important functions of microglia in SCI are phagocytosis (removal of damaged tissue elements), fight against infectious agents, and restoration of homeostasis. The inflammatory response after a traumatic SCI is associated primarily with impairment of the BBB, followed by the release of pro-inflammatory cytokines and activation of adhesion molecules in the vascular endothelium. Next, monocytes, lymphocytes, and macrophages directionally migrate to the injury area [107]. The inflammatory response can lead to local demyelination and apoptosis of neurons. SC microgliocytes quickly respond to trauma: they redirect and extend the cytoplasmic processes in the direction of the lesion and form a dense network that surrounds the injury site. Rapid expansion of microglial processes towards the lesion is mediated by purinergic receptors (P2Y12R), which bind to the ADP released by damaged neurons and astrocytes [110]. During the first days after an injury, microglia are the main type of tissue phagocytic cells; they are in close contact with injured axons, thus utilizing the destroyed myelin. On the third day after a SCI, myelin degradation function is typically largely transferred to macrophages. These cells migrate to the lesion focus and utilize myelin degradation products, which are found in their cytoplasm. Unlike macrophages, resident microglia are found along the periphery of the lesion [111]. Phagocytized material was found to be stored in macrophages for a longer time compared to microgliocytes. This can be explained by the fact that microglial cells more efficiently process disintegrating myelin [112, 113]. The protective role of microgliocytes in a SCI consists in providing conditions for ensuring axon regeneration [114]. The positive effects of microgliocytes appear during the first week after a SCI through the formation of a barrier at the border between the developing connective tissue and the formed astrocytic scar. After a SCI, activated microgliocytes stimulate the proliferation of astrocytes and contribute to the formation of an astrocytic scar, which limits the lesion site. During this period, there is a maximum increase in the number of microglial cells at the site of the injury [115, 116]. Some studies report the ability of microgliocytes to express the leukemia inhibitory factor (LIF), which, in turn, can enhance the survival of oligodendrocytes after a SCI [116, 117]. Other neuroprotective factors, such as the nerve growth factor (NGF), brain-derived neurotrophic factor (BDNF), and glial cell-line-derived neurotrophic factor (GDNF), are also released from microglial cells during this pathological process. However, excessive stimulation of microgliocyte activity can accelerate neuronal damage after a SCI. Activated microglial cells secrete components such as nitric oxide, superoxide, as well as several types of cytokines, including interleukins (IL-1, IL-6) and tumor necrosis factor-α (TNF-α), which can directly or indirectly affect the function of neurons, exert neurotoxic effects, and ultimately lead to neurodegeneration. Intercellular interactions involving immune cells play an important role in the process of recovery after a SCI. A number of studies demonstrate that macrophages isolated from the injured SC actively inhibit the phagocytic activity of microglia and the production of pro-inflammatory cytokines by them, while microglia enhance the phagocytic response of macrophages [113, 118, 119]. Therefore, a potential therapy for SCI can be aimed at modulating the interaction between these cells.

It has recently become clear that not only the presence or absence of microglia or macrophages at the injury site, but also the ratio of their phenotypes allows one to determine the activity of the pathological process in SCI. The balance between pro- and antiinflammatory cell phenotypes is established at the lesion site on day 7 after a SCI. Later, 28 days after injury, microglia and macrophages predominantly express pro-inflammatory markers [112]. An induced shift in the balance of microgliocyte phenotypes towards an anti-inflammatory one may be an effective treatment for SCI [120].

**Microgliocytes and pain**

The pain syndrome is a complex set of symptoms which is most often observed in trauma and inflammation. Damage to the peripheral nerve leads to neuropathic pain and mechanical allodynia, as well as causes extensive microgliosis in the SC. In case of nerve injury, various signaling molecules (caspase-6, neuregulin-1, CXCL1, CSF1, MMP-9, etc.) are released by injured primary afferents, thus causing the activation of SC microgliocytes [121, 122, 123]. Numerous cytokines and chemokines are released by the glial cells alter the transmission of nociceptive information from the periphery to the CNS [124, 125]. Microgliosis caused by nerve damage accompanies the development of pain hypersensitivity, while its inhibition decreases pain behavior. A number of studies show that administration of a non-specific microglia inhibitor to laboratory animals at an early stage after nerve transsection suppresses mechanical hyperalgesia and allodynia [126]. In nerve injury, SC microglia produce BDNF, which induces a change in the penetration of chlorides through the GABAA receptors (GABAAR) in nociceptive neurons. This leads to an increase in their excitability and promotes hypersensitivity to pain stimuli [121, 127]. Microglia can actively regulate the operation of inhibitory synapses by significantly reducing the synaptic presence of GlyR but not GABAAR and thereby reduce the amplitude of spontaneous glycinergic, but not GABAergic, synaptic transmission [128].

It is generally believed that chronic pain is caused by central sensitization, the phenomenon of synaptic plasticity and increased sensitivity of neurons in the central nociceptive circuits that occurs after a pathological event [123]. In hyperalgesia, activated microglia enhance the expression of various cytokines and chemokines, including IL-1β, IL-6, IL-10, TNF, prostaglandin E2 (PGE2), and nitric oxide. As a result, the bioactive molecules released by microgliocytes can potentiate microglia activation through the paracrine mechanism. Moreover, cytokines can also modulate the activity of the neurons and glial cells of the SC dorsal horn during the development of hypersensitivity to pain [123, 129].

Modern studies of visceral pain have revealed the important role of SC microglia in the development of somatic nociception. Microgliocytes show signs of activation during the development of visceral hyperalgesia induced by neonatal colorectal irritation and pancreatitis [129]. Pain syndrome that develops with the involvement of microglia is also observed in conditions such as arthritis, vertebral compression fractures, various types of cancer, as well as in the use of certain medications [122, 123, 130].

According to current research, psychosocial stress and depression contribute to the development of chronic pain [131]. Studies on biological models demonstrated that activation of microglia in the posterior horns of the lumbar SC is accompanied by increased pain sensitivity. On the contrary, elimination of microglia using a colony-stimulating factor 1 receptor antagonist prevents mechanical allodynia. In stress, increased expression of the IL-1β and TNF-α cytokines in lumbar SC and activation of microglia in the regions anatomically associated with the source of pain signals are observed [132]. Thus, there are compelling reasons to believe that SC microglia are a key participant in the pathogenesis of chronic pain.

**Microglia in spinal cord diseases**

Experimental data indicate that dysfunction of the microglia system and an imbalance in its functional states can result in various autoimmune diseases. In the past decade, studying microglia has become the main focus of research in the field of cellular neuroimmunology and, consequently, neuroinflammation. In modern understanding, neuroinflammation is a complex response of nervous tissue to CNS damage, including glia activation, release of inflammatory mediators, and formation of reactive oxygen and nitrogen species [133]. Many of these mediators are produced by activated resident CNS cells, including microglia and astrocytes. Endothelial cells and perivascular macrophages are also involved in the spread of the inflammatory process [134].

Data on the changes in SC microgliocytes in systemic bacterial infections are practically absent. Parenteral administration of lipopolysaccharide (LPS), a component of the outer membrane of gram-negative bacteria, mimics such pathological conditions and triggers a cascade of systemic inflammatory responses [135]. The processes that link the immune response to SC microglia activation remain poorly understood. Microgliocytes are known to express various neurotransmitter receptors. Therefore, neurotransmitters can stimulate microglial cells to trigger a cascade of inflammatory responses or acquire a neuroprotective phenotype [92]. It was noted that, in the gray matter of the SC ventral horns, the thick processes of LPSactivated microglial cells are in close contact with the cytoplasmic membrane of nerve cells in the region of synapses (C-boutons), which is due to their reorganization. Such interactions contribute to changes in neuronal connections during the toxic action of LPS [136]. Some studies report that LPS-induced inflammation can lead to directed migration of circulating monocytes to the CNS [137]. According to other studies, no accumulation of mononuclear cells is observed in the perivascular region a day after the onset of an acute systemic inflammation, which is a typical characteristic of monocyte/macrophage migration. In addition, no round or amoeboid forms of Iba-1-immunopositive cells were detected [136].

SC microgliocytes have a number of important functions in the progression of neurodegenerative diseases. For instance, microgliocytes are actively involved in the pathogenesis of amyotrophic lateral sclerosis (ALS), which is characterized by a loss of motor neurons in the cerebral cortex, brainstem, and the SC. This organic CNS lesion is the most common pathology that leads to impaired functioning of the motor neurons of an adult SC. ALS etiology remains unknown, since most of the cases are sporadic [106]. Perifocal inflammation around motoneurons and axonal degeneration, which is accompanied by the accumulation of reactive astrocytes, activated microglia and lymphocytes, are observed in ALS [138]. An analysis of autopsy material shows that microgliocytes located in the vicinity of damaged neurons express pro-inflammatory markers. The most studied causes of the inherited form of ALS are mutations in the gene *SOD1 *encoding for the superoxide dismutase 1 enzyme [139]. Transgenic mice overexpressing a mutant human *SOD1 *gene (*mSOD*) have a progressive SC pathology similar to ALS. The expression of* mSOD1 *in microglia accelerates the disease onset, while microglia activation causes the death of motor neurons. As the disease progresses, microglia change their phenotype. At the beginning of the disease, microgliocytes isolated from *mSOD1*-carrying mice possess neuroprotective phenotypic properties, in contrast to end-stage microglia [140]. At early stages, the protective function of microglia is realized by limiting damage through phagocytosis of dead neurons and protein aggregates, as well as via the expression of anti-inflammatory and neurotrophic factors. In later periods of the disease, SC microgliocytes exert a neurotoxic effect by activating astrocytes via TNF, IL-1β, IL-6 and by enhancing the inflammatory response, which ultimately leads to neuronal death. The number of pro-inflammatory microgliocytes present in the SC before the onset of clinical signs of the disease increases as the disease progresses and remains in its final stage. Suppression of microgliocyte functions leads to improvement in mice carrying *mSOD1 *[141, 142]. It was established that microglia can attract T cells to a SC lesion, with regulatory T cells (Treg) prevailing in the early stages [106]. At the later stages, their number decreases, while effector T cells prevail [106].

It is worth noting that no *SOD1 *mutations were found in most patients with ALS. Therefore, in order to properly assess disease progression, one should study a neuroinflammation caused by more common pathogenetic factors. Acumulations of cytoplasmic aggregates of the TDP-43 protein are found in the SC of 90% of ALS patients [143]. A study of a biological model of ALS, in which neurodegeneration was caused by TDP-43 overexpression, demonstrated only minor changes in microglia during the development of SC pathology despite the progressive loss of motor neurons. After suppression of *TDP-43 *expression, the number of microgliocytes transiently increases. Moreover, pathological TDP-43 accumulations disappear, which indicates the positive role of microglia in ALS [144].

SC microgliocytes also exhibit predominantly proinflammatory functions in the development of the most common demyelinating disease: multiple sclerosis (MS). It is a chronic neurodegenerative disease characterized by focal inflammatory lesions, microand astrogliosis, intense demyelination of nerve fibers, axon damage, and severe neurological disorders [145, 146]. Currently, the key aspect in the development of inflammation and demyelination in MS is considered to be the penetration of T cells into brain and SC tissues through the disturbed BBB, which leads to the formation of perivascular inflammatory foci. As a result, microglial cells secrete pro-inflammatory cytokines, an increased amount of free radicals and NO in the inflammatory foci, which indicates their key role in the processes of demyelination and neurodegeneration [145]. At the first stage of the disease, microglial cells are activated and localized around inflammatory cell aggregates [147]. At the second stage, the number of activated microgliocytes continues to increase, while the inflammatory foci are also surrounded by the processes of activated astrocytes. During the recovery phase, both microglial and astrocytic gliosis can be clearly identified, and dense astrocyticmicroglial scars start to form [148]. Thus, alongside the involvement of other glial cells, microglia produce an abnormal immune response in multiple sclerosis.

**Spinal cord microglia in aging**

The morphological characteristics and some functions of CNS microglia are known to change with aging [149, 150]. Age-related changes in microgliocytes have been repeatedly noted in brain studies. Such observations regarding the SC are few. However, it is very important to study age-related morphological, phenotypic, and biochemical changes in SC microglia, since these processes can play a significant role in the transmission of pain signals from the periphery to the brain and in the development of the chronic pain syndrome. Understanding the processes that occur in SC microgliocytes during aging will allow one to assess the potential contribution of this cell population to the pathogenesis of age-related sensory disorders.

A number of studies indicate development of abnormal pain behavior in rodents during aging. A study of the population of SC microgliocytes in 17-monthold mice demonstrated that the number of Iba1 immunopositive cells and the proportion of hypertrophied microgliocytes were significantly increased [151]. In addition, filling of the cells with lipofuscin and retraction of microglial processes were observed [151]. Accumulation of lipofuscin indicates both dystrophic changes and pro-inflammatory activation of microglial cells [150]. Indeed, the SC microgliocytes of aging animals exhibit a predominantly pro-inflammatory phenotype [152, 153]. In older animals, activated microgliocytes are localized predominantly in the area of the sensory nuclei of the SC [152]. These facts are of particular importance for understanding the mechanisms of the development of abnormal pain behavior during aging. Age-related shortening and reduction in the branching degree of the processes of microglial cells can lead to the impairment of their ability to control the microenvironment and modulate synaptic activity [151, 154]. Accumulation of lipofuscin by microgliocytes contributes to cellular dysfunction, disruption, and impairment of phagocytic activity [151, 154]. Taken together, these changes can lead to a loss of the neuroprotective potential of microglia, an increase in their neurotoxicity, and a dysregulation of the responses of the SC to sensory signals.

Until recently, the pathogenesis of age-related disorders of skeletal muscle innervation, which results in sarcopenia, an atrophic change in the skeletal muscle leading to a gradual loss of muscle mass, has remained unclear. One of the significant processes in this case is the activation of SC microgliocytes and production of neurotoxic factors disrupting the functioning of motor neurons through them. It has been recently shown that physical activity and selective reduction in microglia population by using an antagonist of the colony-stimulating factor 1 receptor (CSF1R) allow one to preserve motor neurons during aging and eliminate age-related disorders of skeletal muscle innervation [155]. Thus, elimination of the manifestations of neuroinflammation maintained by activated microglia can help in preserving motor neurons and in preventing the development of physical inactivity during aging.

## CONCLUSION

Despite the large number of studies on microglia, many problems related to the biology and functioning of the microglial population of the SC remain controversial and far from resolved. The issues of the interaction between the populations of microgliocytes and macrophages in the SC in various diseases and injuries are also unclear. Most of the studies do not even consider distinguishing between these populations, which undoubtedly exhibit different functional potentials. The classification of microgliocytes also requires unification, with taking into account the distribution of specialized cell types in certain topographic areas of the SC. There is no clarity on the reality of the existence of two (classical and alternative) forms of activated microglia (as in the case of macrophages). More light needs to be shed on the changes in the local and general activity of microglia during aging. The problem related to the functional heterogeneity of microglia, which does not allow for the development of targeted pharmacological agents for preventing neurodegeneration, requires serious investigation. All these factors indicate that it is particularly relevant to develop new methodological approaches that would allow one to conduct experimental and clinical studies of microglia both under physiological conditions and in diseases of the nervous system, as well as in a wide range of pathologies, including endocrine, cardiovascular, and oncological diseases.

## Abbreviations

CNS: central nervous system
SC: spinal cord
DRG: dorsal root ganglion
SCI: spinal cord injury
GABA: gamma-aminobutyric acid
ALS: amyotrophic lateral sclerosis
LPS: lipopolysaccharide
MS: multiple sclerosis
BBB: blood–brain barrier
